# DrugECs: An Ensemble System with Feature Subspaces for Accurate Drug-Target Interaction Prediction

**DOI:** 10.1155/2017/6340316

**Published:** 2017-07-04

**Authors:** Jinjian Jiang, Nian Wang, Peng Chen, Jun Zhang, Bing Wang

**Affiliations:** ^1^School of Electronics and Information Engineering, Anhui University, Hefei, Anhui 230601, China; ^2^School of Computer and Information, Anqing Normal University, Anqing, Anhui 246133, China; ^3^Institute of Health Sciences, Anhui University, Hefei, Anhui 230601, China; ^4^College of Electrical Engineering and Automation, Anhui University, Hefei, Anhui 230601, China; ^5^School of Electrical and Information Engineering, Anhui University of Technology, Ma'anshan 243032, China

## Abstract

**Background:**

Drug-target interaction is key in drug discovery, especially in the design of new lead compound. However, the work to find a new lead compound for a specific target is complicated and hard, and it always leads to many mistakes. Therefore computational techniques are commonly adopted in drug design, which can save time and costs to a significant extent.

**Results:**

To address the issue, a new prediction system is proposed in this work to identify drug-target interaction. First, drug-target pairs are encoded with a fragment technique and the software “PaDEL-Descriptor.” The fragment technique is for encoding target proteins, which divides each protein sequence into several fragments in order and encodes each fragment with several physiochemical properties of amino acids. The software “PaDEL-Descriptor” creates encoding vectors for drug molecules. Second, the dataset of drug-target pairs is resampled and several overlapped subsets are obtained, which are then input into kNN (*k*-Nearest Neighbor) classifier to build an ensemble system.

**Conclusion:**

Experimental results on the drug-target dataset showed that our method performs better and runs faster than the state-of-the-art predictors.

## 1. Introduction

The knowledge of biological targets is key to find new medications where the inventive process is drug design [1]. Most drugs binding to specific proteins activate or inhibit various functions with the change of proteins' biochemical and/or biophysical activities [1–3]. Before designing a drug, some properties of the drug are required to the rational drug design, such as binding affinity, bioavailability, metabolic half-life, and side effects. It is a hard work to obtain these properties accurately. Therefore more attentions are being focused on selecting candidate drugs whose physicochemical properties can be predicted more easier than those of the drug. The former can more likely lead to an approved drug with fast rapid and simplified processes. However, synthesizing a drug candidate involves three issues that should be addressed [1, 2]. First, the way to finding drug effects to different people [3–5] and the biological interaction pathways of the drug effects in human beings [6] are the two important issues. Moreover, since drug discovery is very complicated, the number of new drug approvals is quite low per year. Computational methods are more and more commonly used to design drugs in complement with in vitro experiments, where the identification of drug-target interactions is important in finding potential candidate drugs.

Nowadays, many computational methods have been developed to identify drug-target interactions (DTIs). Chen et al. proposed for the first time a “GO and KEGG enrichment score” method to represent the certain category of drug molecules by constructing a benchmark database [7]. Rarey et al. presented an automatic method for docking organic ligands into protein binding sites, which combined an appropriate model of the physicochemical properties of the docked molecules with efficient methods for sampling the conformational space of ligands [8]. Cheng et al. proposed a model-based approach using basic biophysical principles to predict small-molecule druggability based solely on the crystal structure of target binding sites, which quantitatively estimated the maximal affinity achievable by a drug-like molecule, where the calculated values are correlated with drug discovery outcomes [9]. Zhu et al. proposed a probabilistic model, called mixture aspect model (MAM), with an algorithm for estimating its parameters to mine the relationship of “chemical compound-gene” [10]. Moreover, Chen et al. proposed a prediction method based on Nearest Neighbor Algorithm and a novel metric which combined compound similarity and functional domain composition. It concluded that the combination of compound similarity and functional domain composition is very effective in the drug-target interaction prediction [11].

Some methods combined information of chemical structure, genomic sequence, and 3D structure information to predict drug-target interaction networks [12, 13]. Wang et al. first collected drug pharmacological and therapeutic effects, drug chemical structures, and protein genomic information to characterize the DTIs and then proposed a kernel-based method to predict DTIs by integrating multiple types of data [14]. Other methods developed machine learning methods focusing on HIV protease cleavage site prediction [15], identification of GPCR (G protein-coupled receptors) type [16], protein subcellular location prediction [17, 18], membrane protein type prediction [19], and a series of relevant web-server predictors as summarized in a review [20].

Most recently, researchers proposed many machine learning methods to identify DTIs. Yuan et al. proposed an ensemble method that combined multiple well-known similarity-based methods to predict DTIs [21]. Ba-alawi et al. developed an efficient drug-target prediction method, called DASPfind, that used simple paths of particular lengths inferred from a graph to describe DTIs, similarities between drugs, and similarities between the protein targets of drugs [22]. Moreover, Nascimento et al. proposed a multiple kernel learning algorithm to investigate drug-target bipartite networks and automatically selected the more relevant kernels by returning weights indicating their importance in the drug-target prediction at hand [23]. Liu et al. proposed a neighborhood regularized logistic matrix factorization (NRLMF) for DTI predictions. The NRLMF method modelled the probability that a drug would interact with a target by logistic matrix factorization, where the properties of drugs and targets are represented by drug-specific and target-specific latent vectors, respectively [24]. Recently, Hao et al. proposed a dual-network integrated logistic matrix factorization (DNILMF) algorithm to predict potential DTIs, which encoded drugs and targets by inferring new drug/target profiles, constructing profile kernel matrix, diffusing drug profile kernel matrix with drug structure kernel matrix, and diffusing target profile kernel matrix with target sequence kernel matrix as well [25].

In this work a new prediction system is proposed to identify drug-target interactions. First, drug-target pairs are encoded with a fragment technique and the adopted software “PaDEL-Descriptor.” The fragment technique is for encoding target proteins, which divides each protein sequence into several fragments in order and encodes each fragment with several physiochemical properties of amino acids. The software “PaDEL-Descriptor” creates encoding vectors for drug molecules. Second, the dataset of drug-target pairs is resampled and several overlapped subsets are obtained, which are then input into kNN (*k*-Nearest Neighbor) classifiers to build an ensemble system. Experimental results on the drug-target dataset showed that our method performs better and runs faster than the state-of-the-art predictors.

## 2. Methods

### 2.1. Datasets

To compare with other methods, datasets in [13] were adopted for the drug-target predictions in this study. The datasets contained 4797 drug-target pairs with experimental information, of which 2719 are for enzymes, 1372 for ion channels, 630 for GPCRs, and 86 for nuclear receptors. These datasets are used as the positive ones in this work. To build the drug-target predictor, negative datasets were also selected as in [13]. The selection steps in the reference are (1) separating the pairs in the above positive dataset into single drugs and proteins, (2) recoupling these singles into pairs in a way that none of them occurs in the corresponding positive dataset, and (3) randomly picking the negative pairs thus until they reached the number, two times as many as the positive pairs. The nondrug-target interaction pairs are also divided in terms of protein target family. Finally, the negative datasets contain 9594 drug-target pairs, of which 5438 are for enzymes, 2744 for ion channels, 1240 for GPCRs, and 172 for nuclear receptors. In total, the positive-negative datasets contain 8157, 4116, 1860, and 258 pairs for enzymes, ion channels, GPCRs, and nuclear receptors, respectively.  Table 1 lists the details of the four datasets.

### 2.2. Feature Encoding for Target Protein

**Figure 1:**
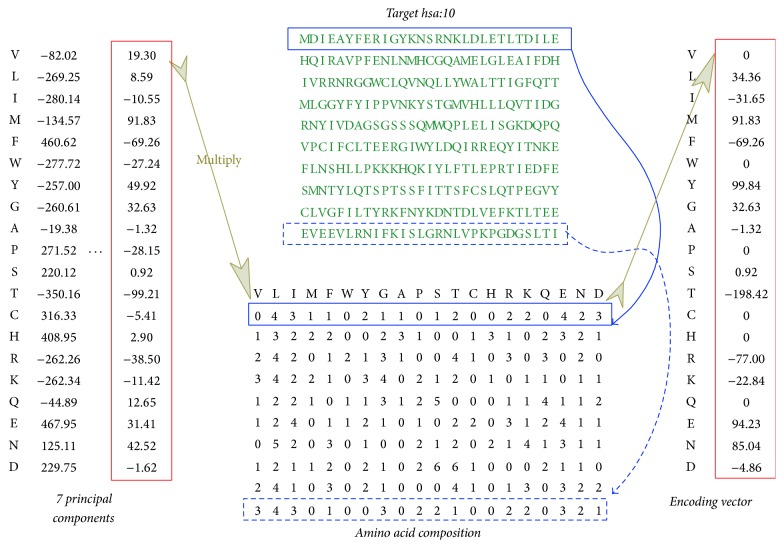
Feature encoding for target proteins. From a target protein, the amino acid composition of segments can be obtained; for example, residue “L” appears four times in the first segment of the sequence and residue “V” three times in the 10th segment.

To encode target protein, 554 physicochemical properties of amino acids from AAindex1 dataset were used. After the use of principal component analysis (PCA), the number of principal components *N*_PCA_ that contribute 97.54% of instance variance is obtained, where *N*_PCA_ = 7 for example. Each principal component of amino acid property is a vector with dimensions 1 × 20, noted as AAP = {AAP_*m*_}, *m* = 1 ~ 20. For target protein TP_*i*_ with sequence length Len, its sequence is divided into *N*_frag_ fragments (denoted as, e.g., *N*_frag_ = 10) with roughly the same numbers of amino acids from the N-terminal to C-terminal of the sequence, that is, INT(Len/*N*_frag_). For *j*th fragment, *j* = 1 ~ *N*_frag_, 20 types of amino acids are counted; that is, AA_*n*_^*j*^, *n* = 1 ~ *N*_AA_, where *N*_AA_ is for 20 types of amino acids. The dot product between the property vector AAP^*l*^, *l* = 1 ~ *N*_PCA_, of *l*th principal component and the amino acid composition AA_*j*_^*n*^ of the *j*th fragment yields the score of amino acid property composition TP_*j*_^*l*,*n*^ = AAP^*l*^⊙AA_*j*_^*n*^. Therefore, the encoding vector for the *j*th fragment is denoted as TP_*j*_ = {TP_*j*_^*l*,*n*^}. Concatenating the ten fragments yields the final encoder vector $\text{T}{\text{P}}^{i}={\left.\left\{\text{T}{\text{P}}_{j}^{l,n}\right\}\right|}_{\begin{array}{ccc} \;l=1 & \;\;j=1 & \;\;n=1 \end{array}}^{\begin{array}{ccc} N_{PCA} & N_{\text{frag}} & N_{\text{AA}} \end{array}}$, where *N*_AA_ means the number of amino acid types *N*_AA_ = 20, for the *i*th whole protein sequence. The vector is then with dimensions 1 × 1400. The encoding scheme for target protein is shown in Figure 1.

### 2.3. Feature Encoding for Drug Molecule

To encode drug molecule, PaDEL-Descriptor software was adopted. “PaDEL-Descriptor” can calculate molecular descriptors and fingerprints for small molecules. Molecular descriptors are the way that transformed the real bodies of molecules into numbers, which can be then evaluated by computer and play a fundamental role in chemistry and other fields. They are traditionally divided into two main categories: experimental measurements and theoretical molecular descriptors. The former category contains log⁡*P*, molar refractivity, dipole moment, polarizability, and physicochemical properties, while the latter one can be derived from a symbolic representation of molecules.

Currently the software creates 1D (i.e., atom count, bond count, chi cluster, and hybridization ratio), 2D (i.e., graph invariants), and 3D (i.e., size, steric, surface, and volume descriptors) molecular descriptors and 10 types of fingerprints [26]. In this work, 55 types of 1D and 2D descriptors were used after removing salt and detecting aromaticity information from a molecule. Among these descriptor types, atom type electrotopological state and autocorrelation are the most significant ones during the used descriptors. The atom type electrotopological state consists of 489 descriptors that combines both electronic and topological characteristics of the analyzed molecules [27]. For each atom type in a molecule, the descriptors are catenated and can be used in a group contribution manner. The type of autocorrelation consists of 346 descriptors that encode not only the structures of molecules but also numerical properties assigned to atoms, proposed by Moreau and Broto [28].

In this study, 1444 descriptors of 1D and 2D types are used to encode drug molecules. So the *i*th drug candidate can be formulated as *D*^*i*^ = [*D*_*j*_^*i*^]^*T*^|_*j*=1_^1444^. For the *i*th pair of drug-target, DT^*i*^, whose target is encoded by the principal components of AAindex1 property AAP, it can be catenated and formulated as a (1444 + 1400)-D input vector given by

(1) Vi,AAPDi,TPiT=D1i,D2i,…,D1444i,TPj,nl l=1  j=1  n=1NPCANfragNaaT,

where *N*_PCA_, *N*_frag_, and *N*_AA_ denote the numbers of top principal components, protein sequence fragments, and amino acid types, respectively. Here *N*_PCA_ = 7, *N*_frag_ = 10, and *N*_AA_ = 20, for example.

The corresponding target value *T*^*i*^ of the instance *V*^*i*,AAP^ is 1 or 0, denoting whether the drug-target pair is in interaction or not. Actually, our method expects to learn the relationship between input matrix *V*^AAP^ and the corresponding target array *T*, and it tries to make the outputs of classifier as close to the target array *T* as possible, where AAP denotes the irrelevant AAindex1 property the targets are encoded by.

### 2.4. Ensemble Classifier by Subspace Seperation of Input Instances

To build a classifier system for the drug-target prediction, the feature space of drug-target instances is separated into several subspaces. For encoding target protein, the use of each principal component of amino acid properties yields features Ft_*i*_, *i* = 1 ~ *N*_PCA_, with dimensions 20 × 10 = 200, while, for drug molecule, approximately 206 (≈1444/*N*_PCA_, *N*_PCA_ = 7) features Fd_*j*_, *j* = 1 ~ *N*_PCA_, are selected in order from the 1D and 2D feature descriptors. A drug-target pair is encoded by one principal component's subfeatures Ft_*i*_ and one subset of 1D and 2D descriptors Fd_*j*_ as $F_{\text{dt}}=\left[\begin{array}{cc} \text{F}{\text{t}}_{i} & \text{F}{\text{d}}_{j} \end{array}\right],\;\;i,j=1\sim N_{PCA}$. There are totally 7 × 7 = 49 feature subsets when *N*_PCA_ = 7, each of which is with dimensions 200 + 206 = 406. The reason of using 7 subsets of 1D and 2D feature descriptors is for balancing the encoding features of target proteins and those of drugs. Pairs of drug-targets with one subset of 406 features are input into classifier system to identify drug-target interactions.

The classifier system adopted kNN algorithm to implement the drug-target prediction. In the case of classification, kNN classifies an object to the most common class among its *k*-Nearest Neighbors by a majority vote, where *k* is a small positive integer. It always assigns weights to the contributions of the neighbors of the object, that is, assigning a weight of 1/*d*, where *d* is the distance between the object and the neighbor. The neighbors are taken from the training set of objects for which the class is known.

Given drug-target pairs (*X*_1_, *Y*_1_), (*X*_2_, *Y*_2_),…, (*X*_*n*_, *Y*_*n*_) taking values in feature space *R*^406^, where *Y* is the class label of *X*, let $F_{\text{dt}}=\left[\begin{array}{cc} \text{F}{\text{t}}_{i} & \text{F}{\text{d}}_{j} \end{array}\right],\;\;i,j=1\sim N_{PCA}$, be the feature subset of drug-target pairs, where *j* and *i* are the subset of drug feature descriptors and that of target features. Training the kNN classifier by drug-target pairs with features *F*_dt_^*i*,*j*^ yields results knn(*X*^*i*,*j*^∣*Y*). After all of the kNN classifiers are generated, they vote for the most popular class and thus the prediction of the ensemble is

(2) $$\begin{array}{c} \text{Pred}\left(\;x\;\right)=\text{majority vote}{\left.\;\left\{\;\text{knn}\left(\;X^{i,j}\;|\;Y\;\right)\;\right\}\;\right|}_{i,j=1}^{N_{PCA}}, \end{array}$$

where *x* is a query instance.

**Figure 2:**
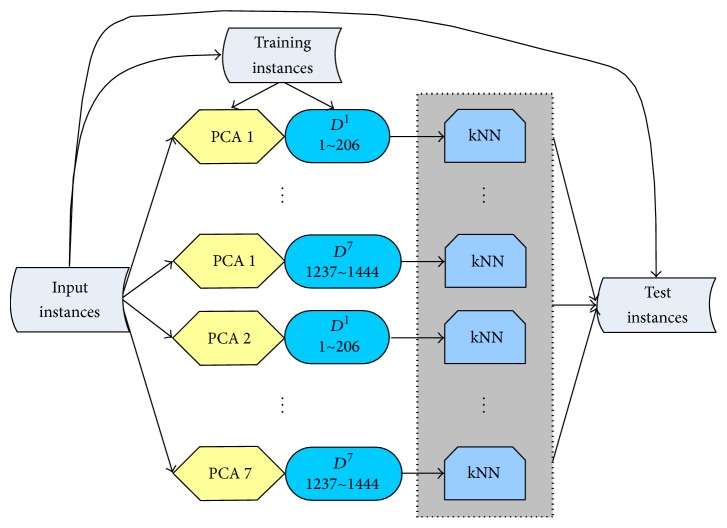
The flowchart of the ensemble system for the drug-target prediction. It illustrates the system for the 7 top principal components. The “PCA 1” denotes the features of protein targets created by the first top principal component, while the “*D*^1^  1 ~ 206” means the first feature group of drugs from 1 to 206 created by the “PaDEL” software. Each feature group consists of the roughly same number of features. Therefore each instance is composed of one “PCA” feature group and one “PaDEL” feature group. In total, there are 49 combinations for the case of the 7 top principal components.

Previous results showed that the majority vote with independent classifiers can often make a dramatic improvement [29, 30]. Here a pair of drug-target is labelled as interacting if all of the classifiers identified it as positive class 1; otherwise it is identified as nondrug-target interaction. The flowchart of the ensemble system can be seen in Figure 2.

### 2.5. Drug-Target Interaction Prediction Evaluation

In this work we adopted four evaluation measures to show the ability of our model objectively, criteria of recall (Rec), precision (Prec), *F*-measure (*F*1), Matthews correlation coefficient (MCC), and accuracy (Acc) [31–33]. They are defined as follows:

(3) Rec=TPTP+FN,Prec=TPTP+FP,Acc=TP+TNTP+FN+FP+FN,MCC=TP×TN−FP×FNTP+FPTP+FNTN+FPTN+FN,F1=2×Prec×SenPrec+Sen,

where TP (true positive) is the number of correctly predicted drug-target pairs, FP (false positive) is the number of false positives (incorrectly overpredicted nondrug-target pairs), TN (true negative) is the number of correctly predicted nondrug-target pairs, and FN (false negative) is false negative, that is, incorrectly underpredicted drug-target pairs.

## 3. Results

In the paper, we adopted kNN algorithm to complete the drug-target interaction predictions. In the use of kNN, for the number of neighbors num_*K*_, we adopted the implementation of WEKA software which used cross-validation technique to find the best number of neighbors. It trained input instances to find which number of neighbors yields the best performance. The trained neighboring number num_*K*_ was then applied to test the test dataset.

Moreover, We used the 7 PCAs in this work because the top 7 components account for the most of instance variance of the 544 amino acid properties, up to 97.54%. The 10 fragments of a protein sequence are adopted corresponding to the number of 1D and 2D descriptors. We used approximately the same numbers of features for drugs and target proteins due to the balance of their effects to classifier. That is to say, if we use 5 fragments of protein sequence, the number of PCAs is suggested to be set as 14 (then the total number of features for protein targets is 5 × 14 × 20 = 1400), 15 fragments are corresponding to 5 PCAs (then the number of features for targets is 15 × 5 × 20 = 1500), and 4 fragments are corresponding to 19 PCAs (1520 features). All numbers of features for targets are approximately the same as the number of features for drugs (totally 1440). For details, please refer to Table 4.

### 3.1. PCA Analysis of the AAindex1 Properties

In AAindex1 dataset, there are 544 amino acid properties. Most of them are highly correlated. In order to extract the main properties from the dataset, PCA technique was adopted. In this study, 7 top principal components are obtained first, which account for 97.54% variance of the properties. Therefore the 7 components are retained and the others are ignored. The other principal components account for only 2.46% variance.  Table 2 shows the 7 principal components.

### 3.2. Performance on Different Top Principal Components of Amino Acid Properties

Drug-target interactions can be commonly grouped in terms of target protein type: enzymes, ion channels, GPCRs, and nuclear receptors. Our proposed method is performed on the four individual types of drug-target interactions. Instances in each interaction type are divided into 10 subsets with roughly the same number of instances, where one subset is used as test dataset *D*_ts_ and the remaining nine subsets are used as training dataset *D*_tr⁡_, by 10-fold cross-validation technique. The test subset is selected one by one and finally all of the instances are tested. Meanwhile, the features of each instance consist of one “PCA” feature group and one “PaDEL” feature group. Inputting the instances into kNN classifier forms a predictor to identify drug-target interactions.

For the case of GPCRs type, the instances encoded by different pairs of drug-target feature groups are input into kNN classifier. The prediction performance can be seen in Table 3. From Table 3, the individual classifier encoded by the third “PCA” group and the seventh descriptor group performs better than others, under the estimation of “Acc,” and the classifiers with the seventh descriptor group perform the best. Moreover, it is interesting to show that the classifiers encoded by the sixth principal component perform better than those by other components.

Also, performance on different numbers of PCAs and the corresponding numbers of fragments is investigated and shown in Table 4. The number of fragments is changed with the number of PCAs since the number of the corresponding features for targets is approximately the same as that of features for drugs (totally 1444 features). Therefore, the instances encoded by the drug features and the target features are used to build drug-target prediction classifier. From Table 4, the model with 7 PCAs performed better than the model with other number of PCAs in terms of the measure *F*1.

### 3.3. Performance of Ensemble System

Table 5 shows the performance comparison of the ensemble system for the four protein target classes. From Table 5, it can be seen that the ensemble system tested on nuclear receptors class performs better than those on other classes. It yields an accuracy of 0.921 and a precision of 0.856 at a recall of 0.916.

### 3.4. Comparison with Other Methods

On the same datasets, our proposed method, called “DrugECs,” was compared with other methods, such as the work in [13], four web-servers, and a random predictor. The performance comparison in terms of the measure “accuracy” is shown in Table 6. Our method yields accuracies of 0.918, 0.882, 0.863, and 0.921 for classes of enzymes, ion channels, GPCRs, and nuclear receptors, respectively. Our method achieves accuracy improvements of 6.3% to 7.8% than the work [13] for the four drug-target classes. Moreover, our method performs better than the four web-servers: iEzy-Drug, iCDI-Drug, iGPCR-Drug, and iNR-Drug. In comparison with the random predictor, the accuracy of our proposed method is increased more than twofold.

**Figure 3:**
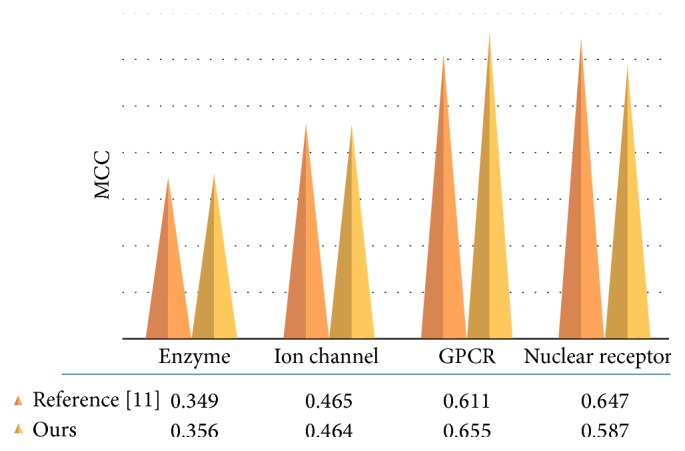
Performance comparison of the two methods in MCC.

Furthermore, we also compared our method with other methods based on another dataset. In [11], Chen et al. proposed a prediction method based on Nearest Neighbor Algorithm and a novel metric combining compound similarity and functional domain composition. It was tested on the database similar to that of our method. The difference between the two datasets is in the fact that, in [11], the number of negative pairs in each target class was around 50 times as many as that of positive ones, while being only 2 times as that of positive ones in our dataset. We compared our method with the work based on the same dataset. Actually, since the dataset is extremely imbalanced, the final performance of drug-target interaction predictions is preferable to the class with large instances, the negative class in this study. The MCC can be a more suitable measure than Acc in the evaluation of classifier performance.  Figure 3 shows the performance comparison of our method and the work in [11]. From Figure 3, our method outperformed the work [11] for the target classes of GPCR and enzyme, and it also performed comparably to the work for the class of ion channel. For the class of nuclear receptor, since the data subset is much small, the prediction performance cannot fully represent the power of methods in drug-target predictions although our method performed worse than the work.

### 3.5. Discussion on the Top 7 Properties

Since the top 7 properties were created by the use of PCA technique on AAindex1 dataset, they account for the main component variance. When calculating the correlation between each top component of PCA and each amino acid property in AAindex1 dataset, the most correlated property in AAindex1 dataset was found for each top component of the PCA calculation.  Table 7 shows the most correlated properties to the components of PCA. Data description of each AAindex1 amino acid property is also shown in Table 7.

In the most correlated AAindex1 properties, two are for hydrophobicity property (the first one and the third one) and two are for position-specific amino acid preferences in helices (the fourth one and the sixth one). Another one is for free energies of transferring amino acid side chains from vapor to cyclohexane that are linearly related to their respective surface areas, which is an experimental measure of their susceptibility to attraction by dispersion forces. The other two properties are the Kerr-constant increments, which can be used in the conformational analysis of peptides and proteins, and the correlation coefficient between the contact areas of residues and their spatial positions from the centroids of the best-fitting ellipsoids. These amino acid properties are important for encoding protein sequence in that they represent protein sequences by different environmental features. The encoding schema aims to apply various statistic features to recover real interactions among amino acid residues.

**Figure 4:**
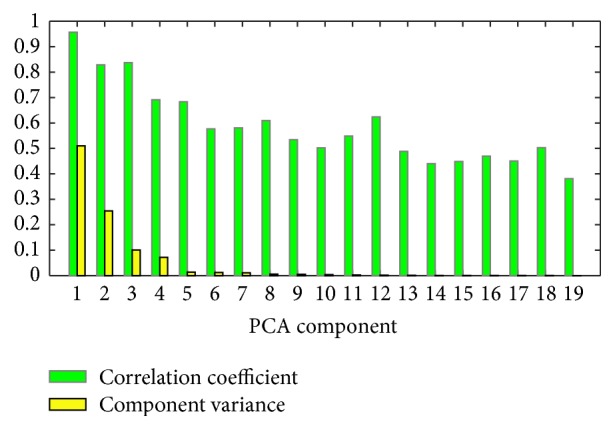
Illustration of the biggest correlation coefficient of each PCA component to AAindex1 properties and the variance of each PCA component accounted for. In *x*-axis, number 1 is for the first principal component while the height of green bar denotes the biggest correlation coefficient between the component and properties in AAindex1 dataset. The yellow bar denotes the variance the component accounted for.

It is interesting to show the correlation coefficient of the top PCA component and AAindex1 property.  Figure 4 illustrates the biggest correlation coefficient of each PCA component to AAindex1 properties and the variance of each PCA component accounted for. From Figure 4, the top PCA component has the biggest correlation coefficient. Moreover, the bigger variance the PCA component contains, the bigger correlation coefficient the component has. It suggests that the trend of the correlation coefficients is in accordance with that of the component variances for the AAindex1 dataset. That is to say, for the case of the first principal component, more properties in AAindex1 dataset can be projected into the component and therefore it is more probable for the component to have bigger correlation coefficient to these properties.

Moreover, the paper proposed a method to identify drug-target interactions only with the physicochemical properties of amino acids from AAindex1. The reason of using physicochemical properties alone is in that it can extend the proposed method to most cases of drug-target predictions. The model with more functional and structural features may lead to better performance than that with only physicochemical properties, but it limited the applications of the structure-based methods without the information of functional and structural features for drug-target predictions.

## 4. Conclusions

This paper proposed an ensemble system integrating kNN classifier with a novel feature encoding scheme to identify drug-target interactions. The features of physicochemical properties from AAindex1 for targets and those of descriptors for drugs are catenated for representing each drug-target pair. The feature space for targets and that for drugs are individually divided into *N*_PCA_ = 7 subspaces in this work. A pair of drug-target feature subspaces forms an input dataset and then is input into a kNN classifier. The total *N*_PCA_ × *N*_PCA_ = 49 feature subspaces lead to 49 individual kNN classifiers. The ensemble system performs the drug-target interaction predictions. Experimental results on the commonly used drug-target dataset showed that our method performs better and runs faster than the state-of-the-art predictors.

## Figures and Tables

**Table 1:** The details of the drug-target dataset.

Dataset	Drugs	Targets	Positive pairs	Negative pairs	Total pairs
Enzymes	419	643	2719	5438	8157
Ion channels	203	198	1372	2744	4116
GPCRs	217	92	620	1240	1860
Nuclear receptors	53	25	86	172	258

In total	892	958	4797	9594	14391^§^

^§^The total number of drug-target pairs in the four datasets.

**Table 2:** PCA physicochemical property.

PCA 1	PCA 2	PCA 3	PCA 4	PCA 5	PCA 6	PCA 7
−82.02	357.18	−55.81	26.80	−18.23	26.74	19.30
−269.25	−276.13	−18.42	137.60	87.32	99.24	8.59
−280.14	−66.50	−85.96	−55.27	−74.70	20.86	−10.55
−134.57	−20.18	243.00	−95.26	−12.48	−25.00	91.83
460.62	102.96	214.88	−209.61	43.97	25.68	−69.26
−277.72	−209.13	−16.25	−51.29	−29.74	2.98	−27.24
−257.00	−101.24	150.56	3.46	−21.27	−7.72	49.92
−260.61	376.97	−105.75	−48.65	90.13	−27.93	32.63
−19.38	−257.49	−154.94	−98.92	26.61	59.15	−1.32
271.52	74.55	−81.98	58.23	−58.45	13.58	−28.15
220.12	203.29	−67.17	179.39	2.03	−26.37	0.92
−350.16	−77.85	212.57	185.66	3.86	−52.35	−99.21
316.33	−140.77	−70.95	−100.15	−67.87	1.04	−5.41
408.95	−30.29	−35.82	67.80	25.37	−5.40	2.90
−262.26	−30.49	−187.34	−151.98	44.89	−97.33	−38.50
−262.34	189.47	−44.11	−28.52	−38.51	40.75	−11.42
−44.89	112.74	155.45	−31.04	−11.86	13.72	12.65
467.95	−270.00	10.58	28.37	52.56	−36.18	31.41
125.11	−178.27	−76.35	104.42	−37.11	−62.19	42.52
229.75	241.19	13.81	78.94	−6.55	36.72	−1.62

51.01%	25.45%	10.09%	7.23%	1.40%	1.26%	1.10%

The last row denotes the variances the components account for.

**Table 3:** Prediction performance on GPCRs dataset for each pair of drug-target feature groups by the use of kNN classifier.

PCA	*D* ^*i*^	Rec	Acc	Prec	*F*1
1	1	0.988	0.349	0.337	0.503
1	2	0.895	0.419	0.353	0.507
1	3	0.988	0.349	0.337	0.503
1	4	0.988	0.357	0.340	0.506
1	5	0.581	0.628	0.455	0.510
1	6	0.930	0.411	0.354	0.513
1	7	0.314	**0.709**	0.628	0.419
Average		0.812	0.460	0.401	0.494

2	1	1.000	0.353	0.340	0.507
2	2	0.895	0.422	0.355	0.508
2	3	0.988	0.345	0.336	0.501
2	4	0.988	0.349	0.337	0.503
2	5	0.570	0.636	0.462	0.510
2	6	0.907	0.426	0.358	0.513
2	7	0.279	**0.717**	0.686	0.397
Average		0.804	0.464	0.411	0.491

3	1	0.988	0.349	0.337	0.503
3	2	0.942	0.391	0.348	0.508
3	3	0.988	0.349	0.337	0.503
3	4	0.988	0.349	0.337	0.503
3	5	0.500	0.632	0.453	0.475
3	6	0.930	0.419	0.357	0.516
3	7	0.419	**0.736**	0.667	0.514
Average		0.822	0.461	0.405	0.503

4	1	0.988	0.349	0.337	0.503
4	2	0.895	0.415	0.352	0.505
4	3	1.000	0.345	0.337	0.504
4	4	1.000	0.349	0.339	0.506
4	5	0.558	0.632	0.457	0.503
4	6	0.942	0.419	0.358	0.519
4	7	0.314	**0.713**	0.643	0.422
Average		0.814	0.460	0.403	0.495

5	1	0.988	0.357	0.340	0.506
5	2	0.895	0.415	0.352	0.505
5	3	0.988	0.349	0.337	0.503
5	4	0.988	0.349	0.337	0.503
5	5	0.570	0.609	0.434	0.492
5	6	0.930	0.407	0.352	0.511
5	7	0.419	**0.686**	0.537	0.471
Average		0.825	0.453	0.384	0.499

6	1	0.988	0.349	0.337	0.503
6	2	0.895	0.426	0.356	0.510
6	3	0.988	0.349	0.337	0.503
6	4	1.000	0.345	0.337	0.504
6	5	0.535	0.640	0.465	0.497
6	6	0.942	0.419	0.358	0.519
6	7	0.337	**0.733**	0.707	0.457
Average		0.812	0.466	0.414	0.499

7	1	0.988	0.349	0.337	0.503
7	2	0.930	0.403	0.351	0.510
7	3	1.000	0.357	0.341	0.509
7	4	0.988	0.349	0.337	0.503
7	5	0.570	0.628	0.454	0.505
7	6	0.965	0.403	0.355	0.519
7	7	0.302	**0.713**	0.650	0.413
Average		0.820	0.457	0.283	0.495

**Table 4:** Prediction performance on GPCRs dataset for different numbers of PCAs and feature groups by the use of kNN classifier.

PCAs	Number of fragments	Number of features^‡^	Rec	Acc	Prec	*F*1
3	24	1440	0.670	0.873	0.930	0.793
5	14	1400	0.914	0.850	0.715	0.801
7	10	1400	0.985	0.863	0.712	0.827
15	5	1500	0.825	0.792	0.648	0.726
19	4	1520	0.631	0.846	0.871	0.732

^‡^The number of features for targets when using the top PCAs and the fragment of protein sequences.

**Table 5:** Prediction performance of the kNN ensemble classifier with majority vote technique. The ensemble system predicts a drug-target pair to be interacting if all of kNN classifiers in the ensemble predict it to be interacting.

Dataset	Target type	Rec	Acc	Prec	*F*1
Test^‡^	Enzymes	0.779	0.918	0.972	0.864
	Ion channels	0.906	0.882	0.778	0.837
	GPCRs	0.985	0.863	0.712	0.827
	Nuclear receptors	0.916	0.921	0.856	0.885

^‡^Prediction on the test dataset *ℵ*_ts_.

**Table 6:** Performance comparison of our method with two works on the same datasets in terms of “Acc” measure.

Method	Type	Enzymes	Ion channels	GPCRs	Nuclear receptors
DrugECs	kNN	0.918	0.882	0.863	0.921
Reference [13]	kNN	0.855	0.808	0.785	0.857
Web-servers		0.910^a^	0.873^b^	0.855^c^	0.892^d^
Random predictor		0.489	0.489	0.488	0.488

^a^See [34] for the iEzy-Drug predictor and its reported success rates; ^b^see [35] for the iCDI-Drug predictor and its reported success rates; ^c^see [36] for the iGPCR-Drug predictor and its reported success rates; ^d^see [37] for the iNR-Drug predictor and its reported success rates.

**Table 7:** The most correlated properties in AAindex1 to the top component of PCA.

PCA	AAindex1	Data description
1	NADH010104	Hydropathy scale based on self-information values in the two-state model (20% accessibility) (Naderi-Manesh et al., 2001)
2	RADA880103	Transfer free energy from vap to chx (Radzicka-Wolfenden, 1988)
3	NADH010107	Hydropathy scale based on self-information values in the two-state model (50% accessibility) (Naderi-Manesh et al., 2001)
4	RICJ880112	Relative preference value at C3 (Richardson-Richardson, 1988)
5	KHAG800101	The Kerr-constant increments (Khanarian-Moore, 1980)
6	RICJ880113	Relative preference value at C2 (Richardson-Richardson, 1988)
7	PRAM820103	Correlation coefficient in regression analysis (Prabhakaran-Ponnuswamy, 1982)

The first column denotes the top components in PCA calculation; the second column denotes the property accessions in AAindex1 dataset.

## Data Access

The data in this study are available at http://www2.ahu.edu.cn/pchen/web/DrugECs.htm.

## References

[1] Johnson D. E., Wolfgang G. H. I. (2000). Predicting human safety: Screening and computational approaches. *Drug Discovery Today*.

[2] Sirois S., Hatzakis G., Wei D., Du Q., Chou K. (2005). Assessment of chemical libraries for their druggability. *Computational Biology and Chemistry*.

[3] Evans W. E., McLeod H. L. (2003). Pharmacogenomics—drug disposition, drug targets, and side effects. *The New England Journal of Medicine*.

[4] Wang J.-F., Wei D.-Q., Chen C., Li Y., Chou K.-C. (2008). Molecular modeling of two CYP2C19 SNPs and its implications for personalized drug design. *Protein and Peptide Letters*.

[5] Wang J.-F., Wei D.-Q., Chou K.-C. (2008). Pharmacogenomics and personalized use of drugs. *Current Topics in Medicinal Chemistry*.

[6] Mizutani S., Pauwels E., Stoven V., Goto S., Yamanishi Y. (2012). Relating drug-protein interaction network with drug side effects. *Bioinformatics*.

[7] Chen L., Chu C., Lu J., Kong X., Huang T., Cai Y.-D. (2015). Gene ontology and KEGG pathway enrichment analysis of a drug target-based classification system. *PLoS ONE*.

[8] Rarey M., Kramer B., Lengauer T., Klebe G. (1996). A fast flexible docking method using an incremental construction algorithm. *Journal of Molecular Biology*.

[9] Cheng A. C., Coleman R. G., Smyth K. T. (2007). Structure-based maximal affinity model predicts small-molecule druggability. *Nature Biotechnology*.

[10] Zhu S., Okuno Y., Tsujimoto G., Mamitsuka H. (2005). A probabilistic model for mining implicit 'chemical compound-gene' relations from literature. *Bioinformatics*.

[11] Chen L., He Z.-S., Huang T., Cai Y.-D. (2010). Using compound similarity and functional domain composition for prediction of drug-target interaction networks. *Medicinal Chemistry*.

[12] Yamanishi Y., Araki M., Gutteridge A., Honda W., Kanehisa M. (2008). Prediction of drug-target interaction networks from the integration of chemical and genomic spaces. *Bioinformatics*.

[13] He Z., Zhang J., Shi X. (2010). Predicting drug-target interaction networks based on functional groups and biological features. *PLoS ONE*.

[14] Wang Y.-C., Zhang C.-H., Deng N.-Y., Wang Y. (2011). Kernel-based data fusion improves the drug-protein interaction prediction. *Computational Biology and Chemistry*.

[15] Chou K.-C. (1993). A vectorized sequence-coupling model for predicting HIV protease cleavage sites in proteins. *Journal of Biological Chemistry*.

[16] Xiao X., Wang P., Chou K.-C. (2009). GPCR-CA: A cellular automaton image approach for predicting G-protein-coupled receptor functional classes. *Journal of Computational Chemistry*.

[17] Chou K.-C., Shen H.-B. (2008). Cell-PLoc: a package of Web servers for predicting subcellular localization of proteins in various organisms. *Nature Protocols*.

[18] Xiao X., Min J.-L., Wang P., Chou K.-C. (2013). Predict drug-protein interaction in cellular networking. *Current Topics in Medicinal Chemistry*.

[19] Chou K.-C., Elrod D. W. (1999). Prediction of membrane protein types and subcellular locations. *Proteins: Structure, Function, and Bioinformatics*.

[20] Chou K., Shen H. (2009). Recent advances in developing web-servers for predicting protein attributes. *Natural Science*.

[21] Yuan Q., Gao J., Wu D., Zhang S., Mamitsuka H., Zhu S. (2016). DrugE-Rank: improving drug-target interaction prediction of new candidate drugs or targets by ensemble learning to rank. *Bioinformatics*.

[22] Ba-alawi W., Soufan O., Essack M., Kalnis P., Bajic V. B. (2016). DASPfind: new efficient method to predict drug–target interactions. *Journal of Cheminformatics*.

[23] Nascimento A. C. A., Prudêncio R. B. C., Costa I. G. (2016). A multiple kernel learning algorithm for drug-target interaction prediction. *BMC Bioinformatics*.

[24] Liu Y., Wu M., Miao C., Zhao P., Li X.-L. (2016). Neighborhood Regularized Logistic Matrix Factorization for Drug-Target Interaction Prediction. *PLOS Computational Biology*.

[25] Hao M., Bryant S. H., Wang Y. (2017). Predicting drug-target interactions by dual-network integrated logistic matrix factorization. *Scientific Reports*.

[26] Yap C. W. (2011). PaDEL-descriptor: an open source software to calculate molecular descriptors and fingerprints. *Journal of Computational Chemistry*.

[27] Kier L. B., Hall L. H. (1990). An Electrotopological-State Index for Atoms in Molecules. *Pharmaceutical Research*.

[28] Moreau G., Broto P. (1980). Autocorrelation of a topological structure: A new molecular descriptor. *Nouveau Journal De Chimie*.

[29] Chen P., Li J. (2010). Prediction of protein long-range contacts using an ensemble of genetic algorithm classifiers with sequence profile centers. *BMC Structural Biology*.

[30] Kuncheva L. I., Whitaker C. J., Shipp C. A., Duin R. P. W. (2003). Limits on the majority vote accuracy in classifier fusion. *Pattern Analysis and Applications*.

[31] Chen P., Li J. (2010). Sequence-based identification of interface residues by an integrative profile combining hydrophobic and evolutionary information. *BMC Bioinformatics*.

[32] Chen P., Liu C., Burge L. (2010). DomSVR: Domain boundary prediction with support vector regression from sequence information alone. *Amino Acids*.

[33] Wang B., Chen P., Huang D., Li J., Lok T., Lyu M. R. (2006). Predicting protein interaction sites from residue spatial sequence profile and evolution rate. *FEBS Letters*.

[34] Min J., Xiao X., Chou K. (2013). iEzy-Drug: A Web Server for Identifying the Interaction between Enzymes and Drugs in Cellular Networking. *BioMed Research International*.

[35] Xiao X., Min J., Wang P., Chou K. (2013). iCDI-PseFpt: Identify the channel–drug interaction in cellular networking with PseAAC and molecular fingerprints. *Journal of Theoretical Biology*.

[36] Xiao X., Min J., Wang P., Chou K., Singh S. (2013). iGPCR-Drug: A Web Server for Predicting Interaction between GPCRs and Drugs in Cellular Networking. *PLoS ONE*.

[37] Fan Y., Xiao X., Min J., Chou K. (2014). iNR-Drug: Predicting the Interaction of Drugs with Nuclear Receptors in Cellular Networking. *International Journal of Molecular Sciences*.

